# 

**Published:** 1890-04

**Authors:** 


### Editorial.

### THE MEDICAL ASSOCIATION OF GEORGIA.

We publish elsewhere the programme of the annual meeting of the Medical Association of Georgia, which will be held in Brunswick on the 16th, 17th and 18th of April. As far as can be judged at present, indications point to a successful and profitable session. It is several years since the Association has met in the Southern part of the State, and it is hoped and confidently expected that many members who have not been in the habit of regularly attending the meetings heretofore, will be induced to be present, and that thus a more general interest in the work of the Association may be awakened. It is to be regretted that so small a proportion of the profession of the State have actively identified themselves with this organization. The advantages to be reaped from such gatherings are very great, and increase in geometrical ratio to the membership and attendance. No active practitioner can afford to forego these advantages or to withhold his aid in furthering the good work. Any temporary sacrifice of time or money devoted to this end, will prove to be bread cast upon the waters which will certainly return after many days.

The programme of the meeting gives evidence of faithful and commendable work on the part of the Secretary, Dr. K. P. Moore, of Macon. It shows that his work has not been merely of a perfunctory character, but that he has the interest of the Association at heart and has labored assiduously for its welfare. Whatever degree of success may attend the Brunswick meeting, will be largely due to his efforts.

We would urge for the approaching meeting, a large and enthusiastic attendance. If every member will make an earnest effort to lend the aid of his presence, and if every one appointed to read a paper or take part in a discussion will do his duty, we believe that this session may be made the beginning of an era of prosperity, such as has never before been known in the history of the Association.

